# A Self-Adhesive Ginsenoside Rk3/Metformin-Loaded Hydrogel Microneedle for Management of Systemic Sclerosis

**DOI:** 10.3390/gels11060384

**Published:** 2025-05-23

**Authors:** Yuanyuan Wang, Caiyun Zhong, Kexin Wang, Shihong Shen, Daidi Fan

**Affiliations:** 1 Engineering Research Center of Western Resource Innovation Medicine Green Manufacturing, Ministry of Education, School of Chemical Engineering, Northwest University, Xi’an 710127, China; wyy110517@163.com (Y.W.); 202332258@stumail.nwu.edu.cn (C.Z.); 2023115192@stumail.nwu.edu.cn (K.W.); 2 Biotechnology & Biomedical Research Institute, Northwest University, Xi’an 710127, China

**Keywords:** systemic sclerosis, hydrogel microneedle, CXCL4/TGF-β, ultra-long release, reverse skin phenotype

## Abstract

Microcirculation damage, dermal thickening, and difficulty in the spatiotemporal coordination of key platelet factor 4 (CXCL4) and transforming growth factor-β (TGF-β) contribute to the lack of effective treatments for systemic sclerosis (scleroderma, SSc). To address these challenges, we proposed a novel synergistic drug combination of ginsenoside Rk3 (CXCL4 regulator) and metformin (Met, TGF-β regulator) based on molecular docking and developed an ultra-long release, dual-target regulation hydrogel microneedle system (Rk3/Met URS MN). The rapidly dissolving tips of this hydrogel microneedle consisted of polyvinyl alcohol and polyvinylpyrrolidone, and were loaded with polydopamine-coated, coordination-induced self-assembled Rk3/Met nanomedicines. These micro-tips could spatiotemporally synchronize transdermal delivery of the hydrophobic Rk3 and hydrophilic Met, providing ultra-long release for up to 10 days with a single administration. The recombinant collagen CF-1552/oxidized pullulan-based (CAOP) hydrogel backing exhibited skin self-adhesiveness and excellent mechanical properties and could perform localized moisture retention and free radical scavenging at the lesion site. In vitro and in vivo efficacy studies, along with bioinformatics analysis of RNA sequencing, demonstrated that the Rk3/Met URS MN achieved immune modulation, anti-inflammatory effects, angiogenesis promotion, and antifibrosis in SSc through synergistic CXCL4/TGF-β dual-target regulation. Notably, on the 10th day, the dermal thickness decreased from 248.97 ± 21.3 μm to 152.7 ± 18.1 μm, with no significant difference from the normal group, indicating its significant potential in clinical applications in SSc.

## 1. Introduction

Systemic sclerosis (scleroderma, SSc) is a chronic, intricate autoimmune connective tissue disorder that currently affects over 1.47 million individuals globally [1,2]. With the potential to manifest at any age, SSc significantly impairs patients’ quality of life and reduces life expectancy [3]. Notably, its mortality rate far exceeds that of many other autoimmune diseases [4]. A particularly concerning aspect is its peak incidence among the prime working-age population, a demographic essential to the labor force [5,6]. This not only disrupts individual lives but also undermines economic productivity and innovation, thereby stymieing societal advancement. Similar to other autoimmune conditions, SSc exhibits a marked female predominance and is often associated with suboptimal prognosis [7,8]. Therefore, the development of a therapeutic agent specifically targeting SSc holds immense scientific and clinical significance.

The pathophysiological mechanisms of SSc remain incompletely elucidated. Genetic predisposition, synergizing with environmental triggers, initiates a pathogenic cascade involving immune dysregulation, inflammation, vascular abnormalities, and aberrant fibrosis, which are established drivers of progressive fibrosis [9,10,11]. Elevated levels of transforming growth factor-β (TGF-β) and platelet factor 4 (CXCL4) in patients are recognized as two key factors positively associated with the occurrence and progression of SSc [12,13]. Specifically, increased CXCL4 levels lead to immune dysregulation, inflammation, and vascular abnormality, while elevated TGF-β levels result in inflammation, vascular abnormality, and skin fibrosis [14,15,16,17]. Thereby, the abnormal overexpression of CXCL4 and TGF-β jointly gives rise to prominent symptoms of SSc, such as skin thickening, hardening, and Raynaud’s phenomenon [18,19]. Consequently, the dual-targeted regulation of CXCL4/TGF-β overexpression-mediated immune dysfunction, inflammation, vascular lesions, and skin fibrosis represents a critical therapeutic imperative of SSc. Established SSc biomarkers include collagen I (Col I) and α-smooth muscle actin (α-SMA) for fibrotic progression, interleukin-6 (IL-6) and tumor necrosis factor-α (TNF-α) for inflammatory activity, platelet endothelial cell adhesion molecule-1 (CD31) for vascular integrity, and anti-centromere antibody (ACA) for systemic autoimmunity [20,21,22,23]. Therefore, regulating the expression levels of these factors through dual-targeted modulation of CXCL4/TGF-β can serve as direct evidence of effective SSc treatment.

However, no synergistic drug combination capable of achieving CXCL4/TGF-β dual-targeted modulation has been reported to date. Currently, clinical approaches aim to alleviate SSc phenotypes by employing glucocorticoids, immunosuppressants, and vasodilators [24,25]. However, this hormone-dependent, “shotgun-style” non-targeted therapy fails to eliminate the core drivers of SSc, offering only short-term relief with limited long-term efficacy [26]. Emerging evidence positions metformin (Met) as a potent TGF-β inhibitor with validated antifibrotic effects in both SSc and diabetic nephropathy models and it has anti-inflammatory and angiogenesis-promoting effects [27,28,29]. Evidently, selecting CXCL4-targeted drugs in combination with Met for the treatment of SSc has the potential for synergistic effects. A large number of previous studies have shown that the natural drug molecules ginsenosides can regulate and treat inflammation, vascular lesions, and immune abnormalities [30,31,32,33]. Given that SSc is characterized by excessive fibrosis, vascular dysfunction, and immune dysregulation, ginsenosides have great potential in treating this disease. The abnormal activation of fibroblasts in SSc patients leads to excessive collagen production, which is consistent with the antifibrotic properties of ginsenosides. In addition, its vascular protection and immune regulatory effects may also help alleviate the complex pathophysiological manifestations of SSc. Theoretical calculations of the binding ability of several rare ginsenosides to CXCL4 through molecular docking have demonstrated that ginsenoside Rk3 is a potential drug for the treatment of SSc. Therefore, Rk3 and Met exhibit a synergistic and complementary regulatory mechanism for the key factors in SSc pathogenesis. The combination therapy of Rk3/Met is expected to become a potential therapeutic option with enhanced synergistic effects on the key target CXCL4/TGF-β in SSc. However, the thickening and hardening of the dermis in SSc skin tissue result in poor drug permeability, rendering transdermal delivery ineffective. Additionally, impaired microcirculation in SSc skin limits the efficacy of systemic drug administration [34]. More importantly, due to the distinct solubility of Rk3 (lipophilic) and Met (hydrophilic), their co-administration in a free state fails to achieve synchronized spatial and temporal effects, significantly reducing the synergistic efficacy of Rk3/Met dual-target regulation of CXCL4 and TGF-β for SSc treatment [35,36,37]. Therefore, improving the targeted delivery efficiency of the Rk3/Met synergistic drug combination to SSc lesion sites is a critical challenge in maximizing its therapeutic efficacy for SSc treatment.

Hydrogel microneedles (MNs) have emerged as transformative transdermal delivery platforms, offering minimally invasive delivery, hepatic first-pass avoidance, and enhanced patient compliance [38,39,40,41,42]. Hydrogel microneedles excel in scleroderma treatment. Their tiny needles penetrate skin non-invasively, delivering drugs to the dermis without damaging the epidermis, thus reducing pain and infection risks. The hydrophilic gel matrix can encapsulate diverse drugs, enabling controlled release and sustained local action. Moreover, hydrogel microneedles’ minimally invasive nature boosts patient compliance, and their mechanical stimulation may enhance microcirculation and remold collagen, potentially relieving skin fibrosis [43,44,45]. Luan et al. fabricated a MN loaded with triptolide and paeoniflorin for SSc therapy, which effectively improved skin transitional fibrosis and telangiectasia in SSc mouse models [46]. The transdermal local drug delivery method based on the MN system has been demonstrated to effectively enhance the therapeutic efficacy of drugs in SSc [47,48]. However, the application of MNs in the multi-target synergistic chemotherapy for SSc has not been reported yet.

**Scheme 1:**
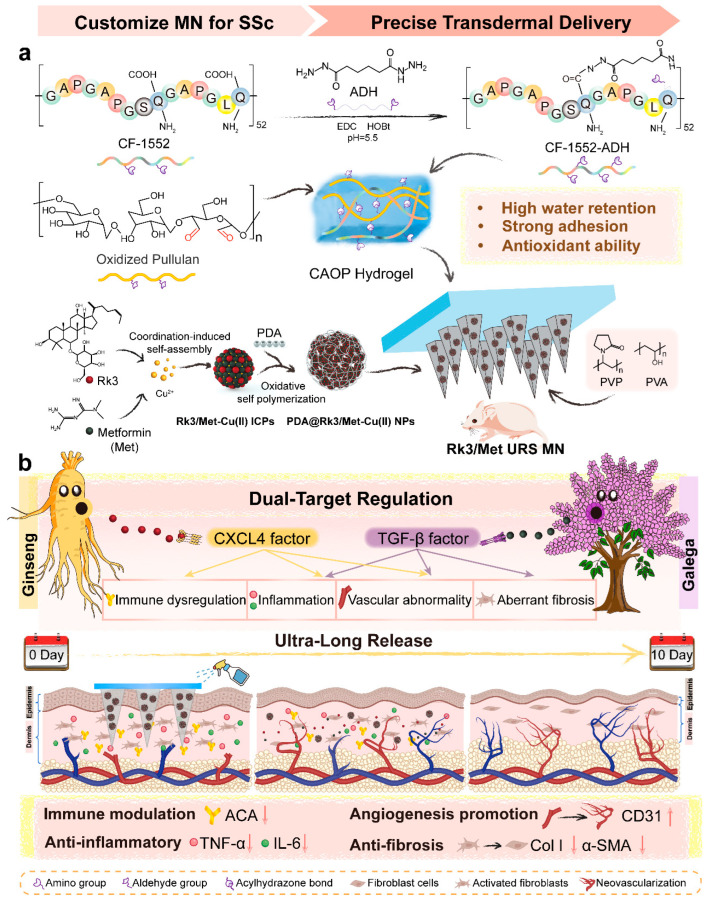
Schematic illustration of the synthesis of the Rk3/Met URS MN (**a**) and the CXCL4/TGF-β dual-target regulation of SSc based on it (**b**).

In this study, a customized ultra-long-release hydrogel microneedle system (Rk3/Met URS MN) was developed for SSc to achieve CXCL4/TGF-β dual-target regulation. The hydrogel backing was formed by dynamically crosslinking ADH-grafted recombinant collagen CF-1552 with oxidized pullulan via imine bonds, exhibiting excellent skin adhesion and mechanical properties while providing localized moisture retention and free radical scavenging. The rapidly dissolving microneedle tips, composed of polyvinyl alcohol (PVA) and polyvinylpyrrolidone (PVP), were loaded with sustained-release polydopamine-coated Rk3/Met nanodrugs, which self-assembled through coordination-induced interactions (Scheme 1a). These microneedles enabled synchronized transdermal delivery of hydrophobic Rk3 and hydrophilic Met, achieving ultra-long drug release with a single administration. Its application in vitro and standardized SSc mouse models demonstrated good biocompatibility, prolonged drug release, and CXCL4/TGF-β dual-targeted immunomodulation, along with anti-inflammatory, pro-angiogenic, and antifibrotic effects. The Rk3/Met URS MN provided an efficient, long-acting, and spatiotemporally synchronized transdermal delivery strategy for Rk3/Met, offering a promising approach for next-generation hydrogel microneedles in targeted SSc therapy (Scheme 1b).

## 2. Results and Discussion

### 2.1. Dual-Target Regulation Synergistic Drug Combination Screening

**Figure 1:**
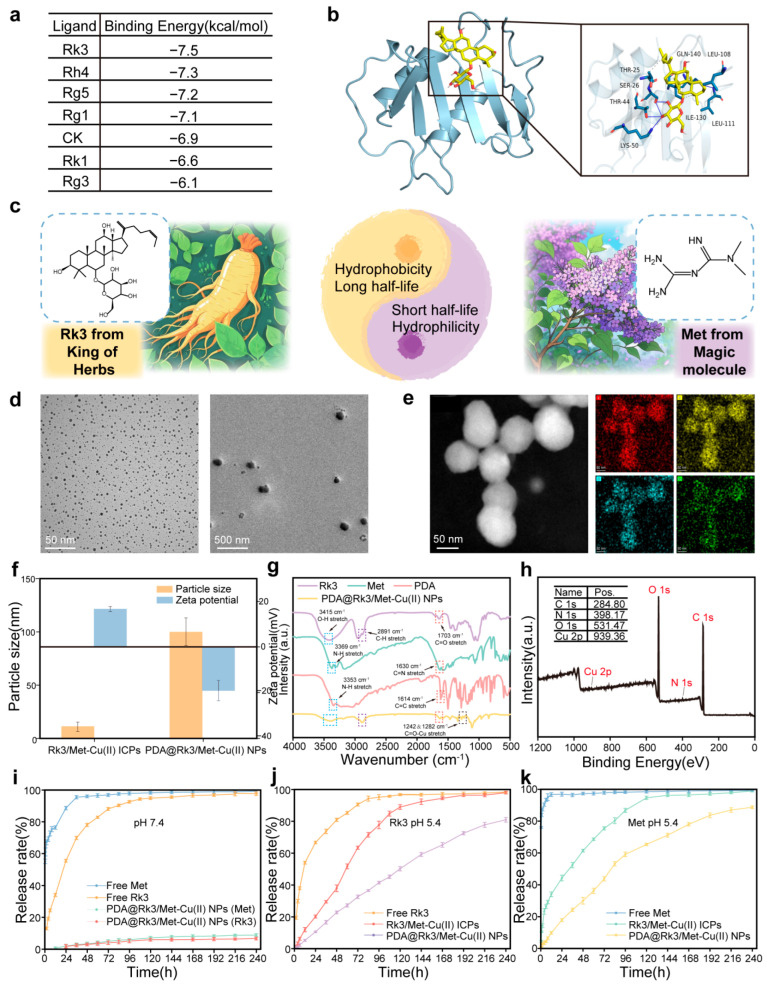
Preparation and characterization of PDA@Rk3/Met-Cu(II) NPs. (**a**) Molecular docking binding energy results of CXCL4 saponin-based targeted regulators. (**b**) Visualization results of the molecular docking between Rk3 and CXCL4. (**c**) Synergistic combination design of Rk3 and Met. (**d**) TEM images of Rk3/Met-Cu(II) ICPs and PDA@Rk3/Met-Cu(II) NPs. (**e**) EDS elemental mapping of C, N, O, and Cu. (**f**) Particle sizes and Zeta potential of Rk3/Met-Cu(II) ICPs and PDA@Rk3/Met-Cu(II) NPs by DLS. (**g**) FT-IR spectrum of PDA@Rk3/Met-Cu(II) NPs. (**h**) XPS spectrum of PDA@Rk3/Met-Cu(II) NPs. (**i**) Cumulative release of Rk3 and Met at pH = 7.4. (**j**) Cumulative release of Rk3 at pH = 5.4. (**k**) Cumulative release of Met at pH = 5.4.

Ginseng is hailed as the “King of Herbs”. Its efficacy against various diseases is attributed to the highly bioactive ginsenoside molecules, especially the rare ginsenosides. A substantial body of previous research had demonstrated that the rare ginsenoside system possesses anti-inflammatory, angiogenesis-promoting, and immunomodulatory effects [49]. The pathway regulation of these effects involves CXCL4, a key target in SSc. The potential pharmacological activity of rare ginsenoside derivatives in CXCL4 modulation has been reasonably postulated based on accumulated experimental evidence. Typical diol-type rare ginsenosides, including Rk3, Rg3, Rk1, and CK, have been reported to have anti-inflammatory, angiogenesis-promoting, and immunomodulatory effects, and typical triol-type rare ginsenosides, such as Rg5, Rh4, and Rg1, have been reported to have anti-inflammatory, cardiovascular-protective, and immunomodulatory effects [50,51]. Molecular docking was performed using AutoDock Vina. The binding energy is an important indicator for measuring the interaction strength between the ligand and the receptor. A lower binding energy indicates a more stable binding and a stronger interaction between the ligand and the receptor [52]. The interaction strengths between the above-mentioned saponin molecules and the CXCL4 receptor protein were scored. The experimental results showed that Rk3 had the lowest binding energy with CXCL4, which was −7.5 kcal/mol (Figure 1a). The docking results were analyzed using PLIP and visualized using PyMOL. As shown in Figure 1b, there were numerous hydrophobic interactions and hydrogen bonds between Rk3 and CXCL4. The hydroxyl groups (-OH) in the Rk3 molecule form hydrogen bonds with the polar groups of amino acid residues (such as serine and threonine) in the CXCL4 protein, which helps to fix Rk3 in the active pocket. The hydrophobic side chains in the Rk3 molecule approach the hydrophobic amino acid residues (such as phenylalanine and leucine) in the CXCL4 active pocket, forming hydrophobic interactions and enhancing the stability of the binding [52]. The results of molecular docking and in vitro preliminary experiment indicated that Rk3 showed promise in targeting and regulating the CXCL4 pathway for SSc treatment. The targeted regulatory effect of the “magic molecule” Met on TGF-β has been studied and confirmed in various disease models, such as SSc and diabetic nephropathy. Yanlin Wang et al. found that metformin significantly reduced the dermal thickness and collagen deposition of SSc by inhibiting the production of TGF-β and promoted vascular stability [27]. Met was selected as the candidate drug molecule combining with Rk3 for the synergistic regulation of CXCL4-TGF-β dual factors.

### 2.2. Preparation and Characterization of PDA@Rk3/Met-Cu(II) NPs

The synergistic regulatory effect of Rk3/Met on the key factors CXCL4/TGF-β in SSc is highly anticipated. However, Rk3 is a hydrophobic drug with poor water solubility (solubility < 0.1 mg/mL), while Met is a hydrophilic drug with a solubility of 50 mg/mL [53,54]. The in vivo half-life of Rk3 is up to several hours, whereas that of Met is only 2 h. The two drugs have significant differences in their in vivo pharmacokinetic pathways and cycles. When administered in their free forms, their spatiotemporal distributions are asynchronous, making it difficult to achieve synergistic effects (Figure 1c). The Cu(II)-coordinated dual-drug-loaded infinite coordination polymers were synthesized, followed by PDA encapsulation to obtain PDA@Rk3/Met-Cu(II) NPs. As shown in Figure 1d, both Rk3/Met-Cu(II) ICPs and PDA@Rk3/Met-Cu(II) NPs exhibited uniform spherical morphologies, with diameters of 1.80 ± 0.04 nm and 90.99 ± 1.79 nm (Figure S1), respectively. By mapping the characteristic signals corresponding to different colors or grayscale regions, the results of EDS elemental mapping showed that the elements C, N, O, and Cu were uniformly distributed on the PDA@Rk3/Met-Cu(II) NPs (Figure 1e). The hydrated particle sizes of Rk3/Met-Cu(II) ICPs and PDA@Rk3/Met-Cu(II) NPs measured by DLS were 10.90 nm and 99.92 nm, respectively (Figure 1f). These results were slightly larger than those obtained by TEM. This was because the TEM observation was conducted under vacuum conditions and excluded the solvent, so the hydrated particle size measured by DLS was larger than that observed by TEM. The surface charge of Rk3/Met-Cu(II) ICPs was 16.7 ± 1.1 mV, while that of PDA@Rk3/Met-Cu(II) NPs decreased to −19.0 ± 4.3 mV (Figure 1f). This was due to the ionization of the catechol groups in PDA and the uneven distribution of the electron cloud under neutral conditions. This change in potential helped to enhance the electrostatic repulsion, thereby improving the dispersion stability of PDA@Rk3/Met-Cu(II) NPs in body fluids. FT-IR analysis results showed that PDA@Rk3/Met-Cu(II) NPs had a broad and strong absorption peak in the 3415 cm^−1^ region, which combined the stretching vibration information of N-H in Met and PDA, and O-H in Rk3. The region at 1615 cm^−1^ contained the contributions of the aromatic ring C=C in PDA, C=N in Met, and C=O in Rk3. Meanwhile, typical metal-coordination absorption peaks of the C=O-Cu group were shown at 1242 cm^−1^ and 1282 cm^−1^ (Figure 1g). In the XPS full-spectrum results, the binding energy peaks of C, N, O, and Cu could be found, which confirmed the chemical composition of PDA@Rk3/Met-Cu(II) NPs in terms of elemental composition (Figure 1h). The above results collectively confirmed the successful preparation of PDA@Rk3/Met-Cu(II) NPs.

Under the condition of pH 7.4, free Rk3 and Met were rapidly released from the dialysis bag through free diffusion, whereas no release of Rk3 and Met was observed from PDA@Rk3/Met-Cu(II) NPs within the 10-day observation period (Figure 1i). PDA@Rk3/Met-Cu(II) NPs exhibited poor drug release behavior under physiological conditions, which was attributed to the polydentate coordination cross-linked chemical structure within the nanodrug [55]. Different from the non-pH-sensitive free diffusion of free Rk3 and Met, under the condition of pH 5.4 (the pH of endosomes is 5.5–6.0 and the pH of lysosomes is 4.5–5.0), Rk3 and Met in PDA@Rk3/Met-Cu(II) NPs could be slowly released within 10 days. Moreover, due to the “cement-caulking-style secondary encapsulation” [56], the release rates of Rk3 and Met from PDA@Rk3/Met-Cu(II) NPs were significantly slower than those from Rk3/Met-Cu(II) ICPs. The release trends of Rk3 and Met were consistent and both reached a release amount of 80% on day 10 (Figure 1j,k). The PDA@Rk3/Met-Cu(II) NPs demonstrated a stimuli-responsive release profile characterized by minimal drug leakage under physiological conditions and sustained release within endo/lysosomal compartments. This pH stimulus-responsive release behavior effectively mitigated the challenges posed by the significant hydrophilic-hydrophobic discrepancy between Rk3 and Met, as well as their differential in vivo metabolic clearance rates. Consequently, the spatiotemporally controlled co-delivery ensured synchronized modulation of dual critical pathogenic factors. The 80% drug release achieved by day 10 enables weekly administration, as compared with daily oral dosing, thus directly enhancing patient adherence by reducing dosing burden. This sustained release profile maintains effective blood concentrations for over 10 days, aligning with the requirement for prolonged target pathway inhibition in SSc and avoiding the peak–trough fluctuations characteristic of conventional therapies. Key advantages include reduced dosing frequency, improved adherence, and consistent target engagement, all of which underscore the clinical practicality of our system.

### 2.3. Preparation and Characterization of Rk3/Met URS MN

**Figure 2:**
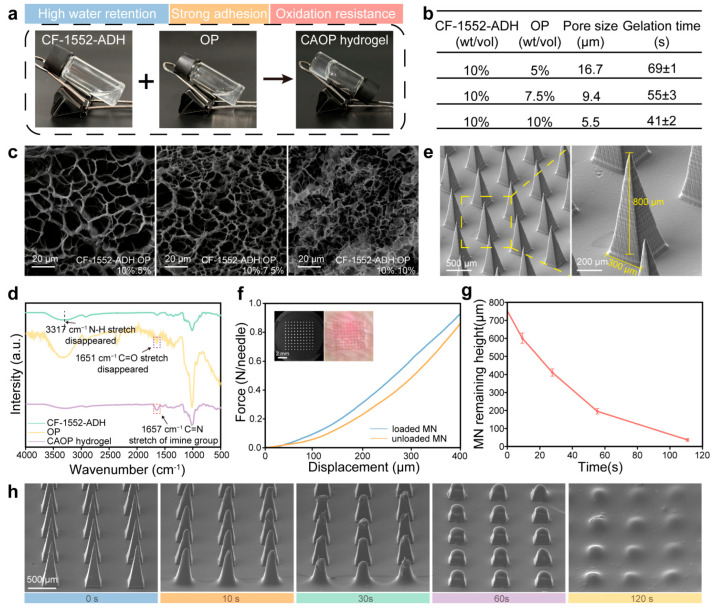
Preparation and characterization of Rk3/Met URS MNs. (**a**) Photograph of the gelation process for CAOP hydrogel backing. (**b**) The pore sizes and gelation times of the CAOP hydrogels with different ratios. (**c**) SEM characterization of different ratios of CAOP hydrogels. (**d**) FT-IR spectrum of CAOP hydrogel. (**e**) SEM characterization of the microstructure of the Rk3/Met URS MNs. (**f**) Mechanical strength tests and transdermal ability tests of the MNs (inset). (**g**,**h**) The remaining heights and SEM images of the dissolved MN tips over time in skin.

To synthesize a hydrogel MN backing with enhanced water retention, strong adhesion, and antioxidant properties, ADH was grafted onto CF-1552 to provide sufficient cross-linking sites for CF-1552 molecules. FT-IR analysis results showed that in the spectrum of CF-1552-ADH, the characteristic absorption peaks of both CF-1552 and ADH could be identified. The absorption peak at 1629 cm^−1^ in CF-1552-ADH was attributed to the C=O stretch of the amide in CF-1552, the peak at 1010 cm^−1^ to the C=N stretch of aliphatic amines in CF-1552, the peak at 2925 cm^−1^ to the C-H stretch of methylene in ADH, and the peak at 3317 cm^−1^ to the N-H stretch of the amide in ADH. These spectral features indicated the successful synthesis of CF-1552-ADH. To form enough acylhydrazone bonds, pullulan was oxidized by NaIO_4_. The stretching vibration peak of C=O in the aldehyde group appeared at 1651 cm^−1^. The emergence of this new characteristic absorption peak confirmed the successful oxidation of pullulan. Meanwhile, pullulan and OP exhibited similar characteristic absorption peaks, suggesting that the original structure of pullulan remained intact and its biological efficacy was unaffected (Figure S2). As shown in Figure 2a, by directly mixing the CF-1552-ADH solution and the OP solution, a CAOP hydrogel could be formed within 1 min. To ensure the effective biological activity of human-like collagen, the total concentration of CF-1552-ADH was fixed at 10% w/v. CAOP hydrogels with different OP concentrations were prepared (Figure 2b). After lyophilization, all hydrogels exhibited an irregular porous network structure (Figure 2c). As the OP concentration was increased, the pore size was decreased and the gelation time was shortened (Figure S3). FT-IR analysis results showed that CAOP hydrogel inherited the characteristic absorption peaks of the CF-1552-ADH and OP skeleton molecules, indicating that the CAOP hydrogel was composed of CF-1552-ADH and OP. Furthermore, the stretching vibration peak of N-H of primary amines in the infrared absorption spectrum of CF-1552-ADH and the stretching vibration peak of C=O in the infrared absorption spectrum of OP disappeared. At the same time, a new stretching vibration peak of C=N appeared at 1657 cm^−1^ in the infrared absorption spectrum of the CAOP hydrogel. These results demonstrated the successful formation of acylhydrazone bonds between CF-1552-ADH and OP (Figure 2d). In conclusion, the CAOP hydrogel was successfully synthesized.

The Rk3/Met URS MNs prepared by a two-step casting method exhibited a complete morphological structure (Figure S4) [46]. As shown in Figure 2e, the MN tips exhibited uniform pyramid-shaped geometries. The Rk3/Met URS MNs featured a 10 × 10 microneedle array (100 tips) with a square configuration, where each microneedle tip exhibited a height of 800 µm and a base diameter of 300 µm. A height of 800 μm was used to ensure complete penetration of thickened SSc skin and promote targeted skin drug delivery. The substrate diameter of 300 μm balanced mechanical strength and scalable manufacturing, minimizing damage while achieving efficient drug loading. The mechanical strength of the MNs was measured through a compression experiment. The results indicated that the mechanical strength of the MNs loaded with nanoparticles was slightly higher than that of the unloaded MNs. When the displacement reached 400 µm, a single MN could withstand a force of over 0.9 N, which was sufficient to penetrate the skin affected by SSc (Figure 2f). A simulation was conducted to study the dissolution of the MNs after piercing the skin. As shown in Figure 2g, the tips of the MNs showed a state of gradual dissolution and almost completely dissolved within 2 min (Figure 2h). This process laid a foundation for the subsequent in vivo function of PDA@Rk3/Met-Cu(II) NPs.

### 2.4. Characterization of the CAOP Hydrogel Backing

**Figure 3:**
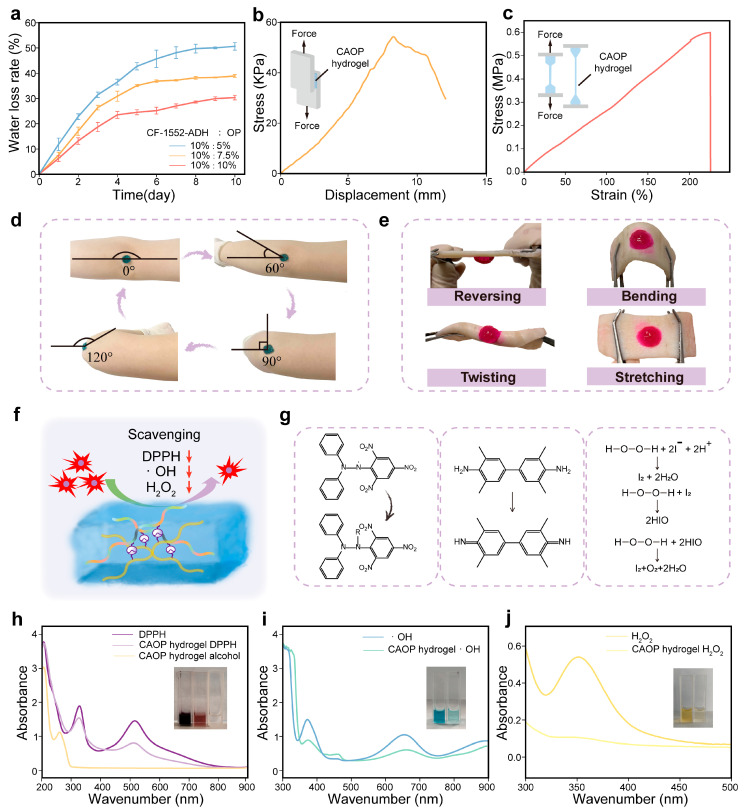
Characterization of CAOP hydrogel backing. (**a**) Water loss rate of CAOP hydrogels with different ratio. (**b**) Displacement–force curve in the lap shear test of the CAOP hydrogel and the schematic diagram of the lap shear test (inset). (**c**) Stress–strain curve of the CAOP hydrogel during stretching. (**d**) Adhesive performance of the CAOP hydrogel on the human elbow during movement. (**e**) The macroscopic mechanical stability of the CAOP hydrogel adhered to porcine skin tissue. (**f**) Antioxidant schematic diagram. (**g**) Mechanism diagram for detecting antioxidant ability. (**h**) Absorbance curves of the CAOP hydrogel before and after the reduction of DPPH. (**i**) Absorbance curves of the TMB color-development method before and after the scavenging of ·OH by the CAOP hydrogel. (**j**) Absorbance curves of the I_2_ color-development before and after the scavenging of H_2_O_2_ by the CAOP hydrogel.

Due to the impaired sweat gland function and excessive proliferation of collagen fibers in SSc skin, the MN backing was expected to possess excellent water retention properties to prevent water evaporation [57]. As shown in Figure 3a, when the proportions of both CF-1552-ADH and OP were 10% w/v, the water loss rate of the CAOP hydrogel backing was the lowest. Therefore, this proportion was selected for subsequent research. The adhesive force of the MN backing to the skin was measured through a lap shear test, and the results showed that the adhesive force reached 53.4 kPa (Figure 3b), which was sufficient to firmly attach it to the skin surface. Furthermore, the CAOP hydrogel backing was designed to possess strong tensile properties, allowing it to closely conform to the skin. As shown in the stress–strain curve, the MN backing exhibited a tensile resistance of 0.6 MPa, allowing it to maintain excellent performance and stability even during skin stretching and deformation (Figure 3c). The hydrogel also exhibited certain adhesive properties and would not fall off during movement (Figure 3d). This was because the unsaturated amino and aldehyde groups on the surface of the CAOP hydrogel could form hydrogen bonds, imine bonds, and hydrazone bonds with proteins, polysaccharides, and other bioactive components in the skin tissue [58]. Moreover, the intuitive tests on the strong adhesion of the CAOP hydrogel demonstrated that it maintained excellent adhesion on porcine skin tissue, even during twisting, stretching, bending, and inversion processes (Figure 3e). The investigation of the adhesion of CAOP hydrogel on porcine skin aimed to validate the robust adhesive capability of the hydrogel microneedle backing layer, demonstrating its ability to withstand external forces and maintain stable adhesion to the skin. This is critical for ensuring sustained functionality in transdermal applications.

The high oxidative stress damage in SSc skin is one of the key triggers that exacerbate the inflammation in its pathological microenvironment. The antioxidant effect of the CAOP hydrogel backing could effectively improve the microenvironment of SSc skin. The main targets of antioxidation are reactive oxygen species (ROS) and reactive nitrogen species (RNS). Typical nitrogen-based radical DPPH, oxygen-based radical ·OH and H_2_O_2_ were employed as model radicals to evaluate the performance of the CAOP hydrogel backing in scavenging RNS and ROS (Figure 3f). The antioxidant ability of the CAOP hydrogel was detected through the following methods [59]. The ethanol solution of DPPH is purple, and it fades when DPPH is reduced to DPPH-H. ·OH can oxidize the colorless TMB DMSO solution to generate a blue solution, with the color depth positively correlated to the concentration of ·OH. A KI solution was used to assess the H_2_O_2_ scavenging ability, where H_2_O_2_ oxidizes I^−^ to iodine (I_2_), turning the solution yellow, with the color depth positively correlated to the H_2_O_2_ concentration (Figure 3g). After DPPH reacted with the CAOP hydrogel, the absorption peak of DPPH at 517 nm decreased and the DPPH solution was significantly decolorized. The scavenging rate for DPPH was 52.9% (Figure 3h). The blue color of the TMB reagent significantly fades after the CAOP hydrogel scavenges ·OH. The peak intensity at 650 nm decreased with a scavenging rate of 57.6% (Figure 3i). The characteristic UV absorption peak of I_2_ is located at 350 nm. The peak intensity of the H_2_O_2_ solution with I_2_ after reacting with the CAOP hydrogel at 350 nm decreased significantly, with a scavenging rate of 90.2% (Figure 3j). The above results indicated that the CAOP hydrogel has good antioxidant ability, which is mainly attributed to the presence of acylhydrazone bonds and the contribution of the amino components in the hydrogel matrix. The above results show that the CAOP hydrogel backing has excellent tissue self-adhesiveness, tensile resistance, water retention, and antioxidant properties during movement.

### 2.5. Biocompatibility and In Vitro Pro-Angiogenic Efficacy

Since the delivery target of the Rk3/Met URS MN is the highly vascularized dermis layer, the blood compatibility of the Rk3/Met URS MN and its components was evaluated. The hemolysis experiment showed no obvious erythrocyte lysis in the Rk3/Met URS MN Tip, CAOP hydrogel, or Rk3/Met URS MN groups, with hemolysis rates below 2%, meeting International Standards Organization standards (<5%) [32]. In addition, the effect of the Rk3/Met URS MN and its components on cell viability was evaluated through co-culture with HUVECs. The test results indicated that, compared with the control group, the cell viability in the CAOP hydrogel group and the Rk3/Met URS MN group increased significantly. There was no significant difference in cell viability between the co-culture group of the Rk3/Met URS MN Tip group and the control group. The results of the cell AO/EB staining supported the above findings (Figure S5). These results suggested that the tip part of the Rk3/Met URS MNs had good biocompatibility and the CAOP hydrogel backing part had a cell-proliferation-promoting effect. This promoting effect may stem from the nutritional support of the degradation products of the CAOP hydrogel skeleton molecules CF-1552-ADH and OP on cell metabolism and the mechanical support of the hydrogel-cell compatible interface [32].

**Figure 4:**
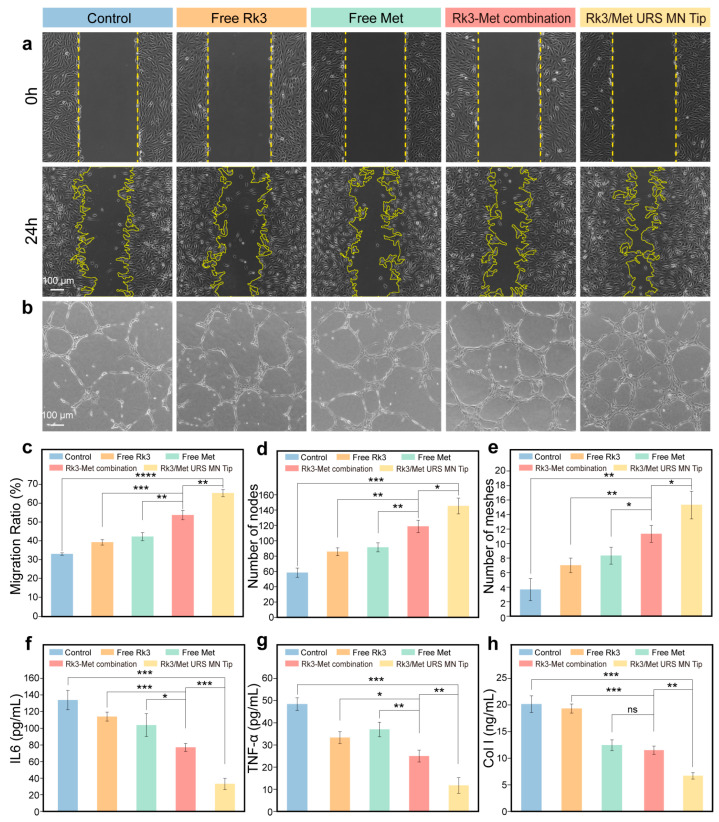
In vitro pro-angiogenic efficacy of Rk3/Met URS MNs. (**a**) Images of HUVECs migration under different treatments. (**b**) Images of tube formation of HUVECs on Matrigel after different treatments. (**c**) Quantitative analysis of cell migration rate. (**d**) Results of the number of nodes in each group. (**e**) Results of the number of meshes in each group. (**f**) ELISA results of IL-6 in HSFs under different treatments. (**g**) ELISA results of TNF-α in HSFs under different treatments. (**h**) ELISA results of Col I in HSFs under different treatments. * *p* < 0.05, ** *p* < 0.01, *** *p* < 0.001, **** *p* < 0.0001.

Breaking the vicious cycle between the ischemic and hypoxic state of the lesion caused by the reduction of skin microvessels and insufficient skin blood supply and the poor drug response and treatment resistance of the SSc skin phenotype is one of the goals of SSc treatment [60]. The effect of Rk3/Met URS MN on the migration of HUVECs under a simulated SSc environment (10 ng/mL TGF-β and CXCL4, respectively) was evaluated by the scratch assay [61]. Compared with the migration rate of 32.9% in the control group, the migration rates in the free Rk3 group, the free Met group, and the free Rk3-Met combination group were 39.2%, 42.2%, and 53.7%, respectively. This indicated that both Rk3 and Met have the effect of promoting HUVECs’ migration and their combination has a synergistic promoting effect. The migration rate of HUVECs in the Rk3/Met URS MN Tip group was significantly increased to 65.3%, suggesting that the loaded PDA@Rk3/Met-Cu(II) NPs can improve the drug utilization rate (Figure 4a,c).

In vitro angiogenesis tube formation experiments were performed also under the simulated SSc environment to further quantify the in vitro pro-angiogenic efficacy of Rk3/Met URS MN at the cellular level. As shown in Figure 4b, compared with the control group, the free drug groups, and the free drug combination group, the Rk3/Met URS MN Tip group exhibited significantly higher numbers of nodes, meshes, total mesh area, and total tube lengths (Figure 4d,e and Figure S6). The quantitative results of tube formation by HUVECs showed that the number of nodes in the Rk3/Met URS MN Tip group was 2.2-fold that of the control group and 1.22-fold that of the free dual-drug combination group; the number of meshes was 3.75-fold that of the control group and 1.36-fold that of the free dual-drug combination group; the total mesh area was 2.61-fold that of the control group and 1.31-fold that of the free dual-drug combination group; and the total tube length was 1.4-fold that of the control group and 1.12-fold that of the free dual-drug combination group (Figure S7). All the above differences were statistically significant. The above experimental results indicated that the combination of Rk3 and Met has excellent in vitro pro-angiogenic ability and the multi-layer encapsulation and delivery of Rk3/Met URS MN can effectively amplify this synergistic effect.

### 2.6. In Vitro Anti-Inflammatory and Anti-Fibrotic Efficacies

Anti-inflammation alleviates the symptoms of SSc and slows down the progression of the disease by reducing inflammatory damage, suppressing immune abnormalities, and improving vascular function. Anti-fibrosis, on the other hand, reduces the degree of tissue fibrosis and improves organ function by inhibiting fibroblast activation and promoting collagen degradation. Both play crucial roles in the treatment of SSc [62,63]. The levels of inflammatory factors and type I collagen in fibroblasts of the HSFs under the simulated SSc environment were evaluated after treatment with Rk3/Met URS MN and its components. The results of the ELISA showed that, compared with the control group, both the free Rk3 group and the free Met group exhibited good inhibitory efficacies on the inflammatory factors IL-6 and TNF-α (Figure 4f,g). The inhibitory efficacy was more pronounced in the free Rk3-Met combination group, indicating a synergistic effect between Rk3 and Met. The Rk3/Met URS MN Tip group showed the most significant inhibitory efficacy, suggesting that the nanonization could improve drug utilization, enhance drug delivery capacity, and strengthen drug efficacy [64]. Culturing in a simulated SSc environment with CXCL4 and TGF-β concentrations leads to excessive activation of HSFs and excessive Col I deposition. The experimental results of reducing Col I deposition at the cellular level indicated that Met was the key agent responsible for inhibiting Col I deposition. The Rk3/Met URS MN Tip group showed a significantly enhanced inhibitory efficacy on Col I deposition, further demonstrating that the loaded nanodrug could improve drug utilization, enhance drug delivery capacity, and strengthen drug efficacy (Figure 4h). These findings provided cellular-level evidence that the Rk3/Met URS MN Tip group exerted notable anti-inflammatory and anti-fibrotic efficacies in a simulated SSc environment.

### 2.7. In Vivo SSc Therapy Efficacy of Rk3/Met URS MNs

**Figure 5:**
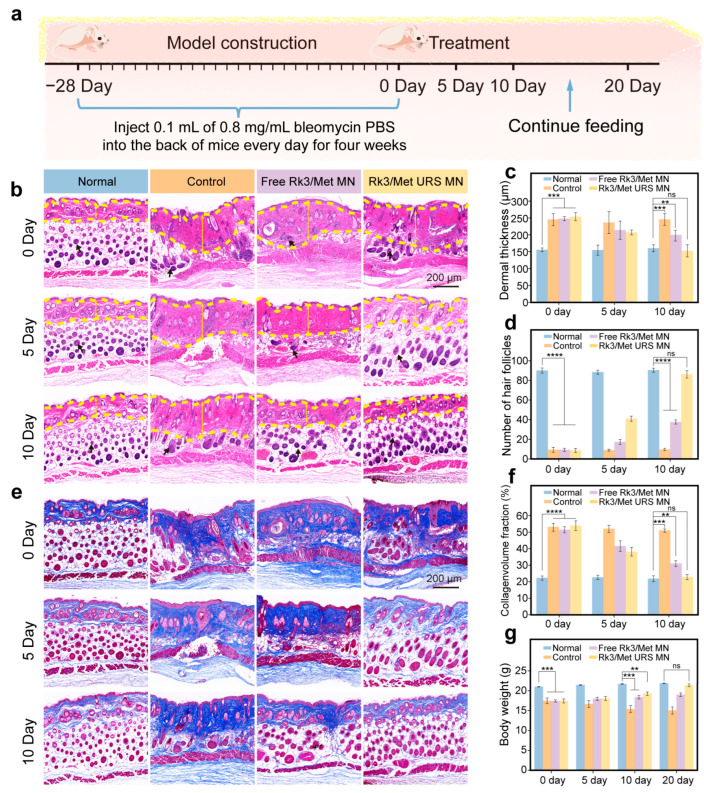
In vivo therapeutic efficacy of Rk3/Met URS MNs in SSc. (**a**) Construction of the SSc mouse model and treatment schedule. (**b**) H&E staining of mouse skin from each group over time. (**c**) Quantitative analysis of dermal thickness in each group. (**d**) Number of hair follicles in mice. (**e**) Masson staining of mouse skin from each group over time. (**f**) Quantitative analysis of collagen deposition in each group. (**g**) Changes in body weight of mice in each group over time. ** *p* < 0.01, *** *p* < 0.001, **** *p* < 0.0001, ns non-significant.

A classic bleomycin PBS solution was used to induce the establishment of SSc mouse models (Figure 5a). Specifically, during the establishment of SSc mouse models, 0.1 mL of 0.8 mg/mL bleomycin PBS solution was injected daily for the first 28 days, and the mice were weighed regularly [23]. Following the model construction, the murine skin exhibited a notable hardening. In the H&E staining skin sections, the dermal thickness reached 248.97 ± 21.3 μm, which was 1.6-fold that of the normal group. Concurrently, Masson staining revealed abnormal collagen deposition within the skin. In addition, the body weight of the modeled mice decreased significantly compared with that of the normal group (Figure S8).

Four groups were established: normal, control, free Rk3/Met MNs, and Rk3/Met URS MNs, with 18 SSc mice randomly assigned to each group. On day 0, free Rk3/Met MNs and Rk3/Met URS MNs were inserted into the back skin of the mice, and six mice in each group were sacrificed on day 5 and day 10, respectively (Figure 5a). The skin at the lesion site was sectioned and observed by H&E staining. The results showed that there was no obvious change in the SSc phenotype of the mice in the control group within 10 days of placebo treatment, indicating that SSc cannot spontaneously alleviate without drug treatment. The dermal thickness of the skin in both the free Rk3/Met MN group and the Rk3/Met URS MN group decreased significantly compared with that in the control group (Figure 5b). The quantitative results of the dermal thickness of the mouse skin (the dermis layer is indicated by the yellow arrow) showed that after 10 days of treatment, the dermal thickness in the Rk3/Met URS MN group was 152.7 ± 18.1 μm, showing no significant difference from that in the normal group. In contrast, the dermal thickness in the free Rk3/Met MN group was 189.8 ± 16.8 μm and the efficacy of reducing dermal thickness was significantly weaker than that in the Rk3/Met URS MN group (Figure 5c). Statistical results of the temporal changes in the number of hair follicles in the mouse skin showed that the number of hair follicles in the control group decreased significantly compared with that in the normal group. In the Rk3/Met URS MN group, the number of hair follicles recovered to 41 ± 3 on day 5 (the number of hair follicles in the normal group was 90 ± 2), achieving a stage-wise therapeutic efficacy. On day 10, there was no significant difference in the number of hair follicles compared with the normal group (Figure 5b,d, where the hair follicles are indicated by the black arrow). Masson staining results indicated that during the treatment period, collagen deposition in both the free Rk3/Met MN group and the Rk3/Met URS MN group decreased significantly compared with that in the control group, and the decrease was more obvious in the Rk3/Met URS MN group (Figure 5e). The quantitative results of collagen deposition showed that there was no significant difference in skin collagen deposition between the Rk3/Met URS MN group and the normal group on day 10, while there was still a significant difference between the free Rk3/Met MN group and the normal group (Figure 5f). During the drug-administration process, sections of the heart, liver, spleen, lungs, and kidneys of the mice were stained with H&E. The results showed no obvious pathological changes in these organs, indicating that local administration of Rk3/Met at this dose caused no significant organ toxicity (Figure S9). In addition, body weight monitoring during the treatment showed that the body weight of the mice in the control group decreased significantly, the decreasing trend of body weight in the free Rk3/Met MN group was weakened, and the body weight of the mice in the Rk3/Met URS MN group started to increase. On day 10, the difference in body weight from the normal group was reduced to 2.43 ± 0.29 g, and this difference disappeared when the feeding time was extended to day 20 (Figure 5g). It was observed that the hair on the back of the mice resumed growth under the treatment of the Rk3/Met URS MNs (Figure S10). The above results indicated that Rk3/Met URS MNs could effectively reduce dermal thickness, inhibit abnormal collagen deposition in the dermis, restore the structure of skin appendages, and reverse the weight-loss trend in SSc mice.

### 2.8. In Vivo Dual-Target Regulation of CXCL4/TGF-β Using Rk3/Met URS MNs

**Figure 6:**
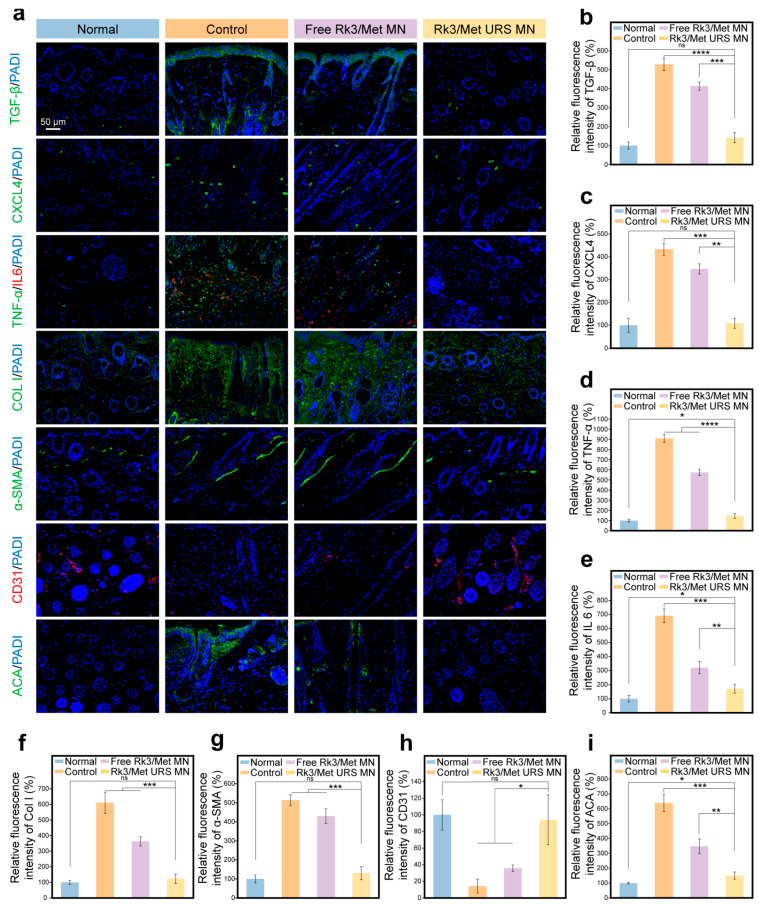
In vivo dual-target regulation of CXCL4/TGF-β using Rk3/Met URS MNs. (**a**) Staining results of various biological indicators. (**b**) Quantitative analysis of relative expression levels of TGF-β. (**c**) Quantitative analysis of relative expression levels of CXCL4. (**d**) Quantitative analysis of relative expression levels of TNF-α. (**e**) Quantitative analysis of relative expression levels of IL-6. (**f**) Quantitative analysis of relative expression levels of Col I. (**g**) Quantitative analysis of relative expression levels of α-SMA. (**h**) Quantitative analysis of relative expression levels of CD31. (**i**) Quantitative analysis of relative expression levels of ACA. * *p* < 0.05, ** *p* < 0.01, *** *p* < 0.001, **** *p* < 0.0001, ns non-significant.

First, the expression levels of two key factors, CXCL4 and TGF-β, in the diseased tissues regulated by Rk3/Met URS MNs were evaluated. Experimental results showed that the levels of CXCL4 and TGF-β in the Rk3/Met URS MN group decreased significantly compared with those in the control group and there was no significant difference in CXCL4 and TGF-β levels between the Rk3/Met URS MN group and the normal group (Figure 6a–c). Moreover, the Rk3/Met URS MN group outperformed the free Rk3/Met MN group. This was because the nano-formulation of PDA@Rk3/Met-Cu(II) NPs offers “spatiotemporal synchronization” of drug release and ultra-long release, which were superior to the free administration of Rk3 and Met. These advantages amplified the synergistic effect of the combined drugs.

Further evaluation was conducted on inflammation, skin fibrosis, immunity, and angiogenesis regulated by CXCL4 and TGF-β to explore the process and biological mechanisms of lesion repair. The high expression of TNF-α and IL-6 in SSc skin fibroblasts is the root cause of the high-grade inflammatory response in SSc. Double immunofluorescence staining for TNF-α and IL-6 was performed on the lesional tissue on day 10 to assess the inflammation intensity at the lesion site [20]. As shown in Figure 6d,e, compared with the control group and the free Rk3/Met MN group, the expression levels of TNF-α and IL-6 in the Rk3/Met URS MN group were significantly reduced, indicating the remarkable anti-inflammatory activity of Rk3/Met URS MNs in the SSc model. Immunofluorescence staining of COL I and α-SMA showed a decrease in collagen expression and a reduction in α-SMA expression in the collagen layer, respectively. There was no significant difference in the levels of COL I and α-SMA on day 10 between the Rk3/Met URS MN group and the normal group, suggesting that Rk3/Met URS MNs could significantly inhibit the fibrotic effect of SSc, and the abnormal collagen deposition in the skin tissue after MNs treatment was maximally alleviated (Figure 6f,g). CD31 is a marker for neo-vascular endothelium. No CD31 fluorescence signal was observed in the SSc skin tissue of the control group, while the CD31 fluorescence signal was significantly increased in the Rk3/Met URS MN group (Figure 6h). High expression of the immune marker ACA can reflect the abnormal immune status of the SSc body [20]. ACA immunofluorescence staining showed a significant decrease in ACA expression levels in the Rk3/Met URS MN group compared with the control group on the 10th day after treatment (Figure 6i), indicating that the abnormal immune response in SSc skin lesions was effectively regulated during this treatment period. The above results indicated that Rk3/Met URS MNs had an ideal therapeutic efficacy on the four key issues of early-stage SSc immune dysregulation, inflammation, vascular abnormality, and aberrant fibrosis under the regulation of the dual key factors CXCL4/TGF-β.

**Figure 7:**
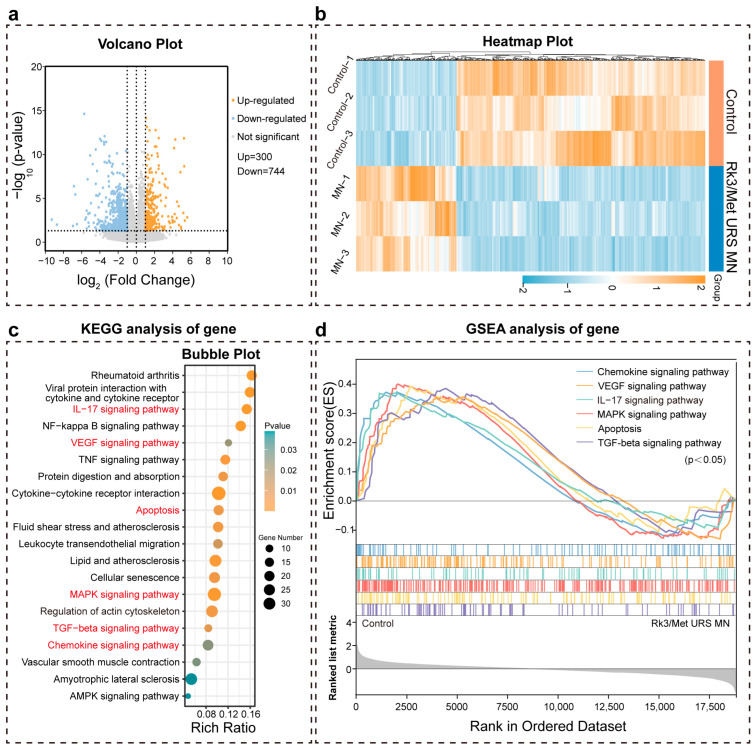
Bioinformatics analysis of RNA-seq for control and Rk3/Met URS MN groups. (**a**) Volcano plot comparing differentially expressed genes. (**b**) Heatmap showing the differential gene expression. (**c**) Bubble plot of KEGG pathway analysis. (**d**) Results of GSEA.

Bioinformatics analysis of RNA-seq data from the diseased tissue was performed to further investigate the possible signaling pathways involved in the regulation of CXCL4 and TGF-β key factors. The results showed that there were 1044 differentially expressed genes in the Rk3/Met URS MN group compared with the control group (*p* < 0.05, Log_2_ fold change > 1 or <−1) (Figure 7a). Among them, 300 genes were up-regulated and 744 genes were down-regulated (Figure 7b). KEGG pathway analysis of these differentially expressed genes revealed multiple significantly enriched pathways, which were involved in four different types of biological processes: immune dysregulation, inflammation, vascular abnormality, and aberrant fibrosis (Figure 7c). The MAPK signaling pathway was involved in the fibrosis and inflammation processes of SSc. Abnormal activation of this pathway led to the proliferation of fibroblasts and excessive production of collagen. The NF-kappa B signaling pathway played an important role in inflammation and immune responses [65]. Activation of this pathway in SSc results in persistent inflammation and fibrosis. Cytokines such as IL-6 and TNF-α played important roles in the pathogenesis of SSc. These cytokines interact with their receptors to promote inflammation and skin fibrosis. The VEGF signaling pathway was involved in abnormal angiogenesis and endothelial cell dysfunction in SSc, leading to vascular lesions. Meanwhile, activation of this pathway causes immune abnormalities and skin fibrosis [66]. The IL-17 signaling pathway played a key role in the pathogenesis, disease progression, and tissue fibrosis of SSc through mechanisms such as inducing inflammatory responses, activating and regulating immune cells, promoting fibrosis, and affecting the function of vascular endothelial cells [67]. The results of gene set enrichment analysis (GSEA) supported that genes related to the positive regulation of inflammation, skin fibrosis, immunity, and angiogenesis were significantly down-regulated in the Rk3/Met URS MN group, which was consistent with the results of KEGG pathway analysis (Figure 7d). Notably, the chemokine signaling pathway and TGF-beta signaling pathway corresponded exactly to the CXCL4 and TGF-β pathways. In conclusion, problems such as inflammation, skin fibrosis, immunity, and vascular lesions caused by SSc could be effectively restored by the treatment of Rk3/Met URS MNs. Based on the above research, Rk3/Met URS MNs are a promising treatment for SSc.

## 3. Conclusions

We proposed a novel Rk3/Met synergistic drug combination and developed an ultra-long-release hydrogel microneedle system (Rk3/Met URS MNs) for SSc therapy. These hydrogel MNs feature CXCL4/TGF-β dual-target regulation, addressing key challenges of existing therapies, including poor transdermal penetration, limited multi-system therapeutic efficacy, and high dosing frequency leading to low patient compliance. The Rk3/Met URS MNs demonstrate excellent mechanical properties, with each MN sustaining forces over 0.9 N, effectively penetrating thickened SSc skin and dissolving within 120 s to release PDA@Rk3/Met-Cu(II) NPs. These nanoparticles enable in situ ultra-sustained Rk3/Met release for over 10 days, ensuring synchronized spatiotemporal CXCL4/TGF-β dual-target regulation. Additionally, the rehydrated CAOP backing self-adheres to the skin (adhesion strength: 53.4 kPa), remains intact during skin stretching, and actively scavenges reactive oxygen and nitrogen species. In vitro and in vivo studies demonstrated that the Rk3/Met URS MNs effectively regulate the four hallmark pathological features of SSc, including immune dysregulation, inflammation, vascular abnormality, and aberrant fibrosis, by down-regulating ACA, TNF-α, IL-6, Col I, and α-SMA while simultaneously up-regulating CD31. Notably, within only 10 days, the dermal thickness decreased from 248.97 ± 21.3 μm to 152.7 ± 18.1 μm, with no significant difference from the normal group. Compared with existing treatments, our innovative platform demonstrates unparalleled efficacy, completely reversing the phenotype of scleroderma skin. It also exhibits excellent biocompatibility, ensuring minimal adverse reactions. Notably, it reduces the dosing frequency to one-tenth of that required by conventional therapies, enhancing patient convenience and compliance. These compelling results highlight the remarkable potential of our hydrogel system as an innovative therapeutic approach for SSc, offering new avenues for enhancing patient outcomes and improving surgical interventions. By integrating dual-target nanotherapeutics with adaptive material engineering, this work opens new avenues for developing next-generation transdermal platforms—ones that not only treat existing fibrotic lesions but also preemptively modulate pathological pathways, redefining the landscape of SSc management and offering a blueprint for translating nanomedicine innovations into clinical practice. In future perspectives, although our study demonstrates that Rk3 and Met regulate scleroderma pathology, the underlying signaling mechanisms remain unclear. Future research employing gene editing and multi-omics will clarify how nanoparticles modulate pathway interactions to optimize combinatorial therapies. To address complex comorbidities, investigations into organ-specific interventions using advanced models are needed to improve clinical outcomes.

## 4. Materials and Methods

### 4.1. Materials

Human-like collagen CF-1552 and ginsenoside Rk3 were procured from Shaanxi JUZI Biology Gene Technology Co., Ltd. (Xi’an, China). Adipic dihydrazide (ADH), 1-hydroxybenzotriazole (HOBt), 1-(3-dimethylaminopropyl)-3-ethylcarbodiimide hydrochloride (EDC), and sodium periodate (NaIO_4_) were obtained from Sigma-Aldrich (St. Louis, MO, USA). All experimental protocols involving animals were approved by the Laboratory Animal Management and Welfare Ethics Committee of Northwest University.

### 4.2. Molecular Docking Screening of Rare Ginsenosides

The three-dimensional structure of CXCL4 was retrieved from the Protein Data Bank. Ligand structures for rare ginsenosides (Rk3, Rh4, Rg5, Rg1, CK, Rk1, and Rg3) were constructed using ChemBio3D Ultra 14.0. Molecular docking was performed in AutoDock 4.2.6 and PyMOL 2.5.2 was used to select the lowest energy conformation for interaction analysis.

### 4.3. Preparation of PDA@Rk3/Met-Cu(II) NPs

First, 500 µL of a 3 mg/mL Rk3 alcohol solution was combined with 500 µL of a 3 mg/mL Met alcohol solution. Subsequently, 20 µL of a 10 mg/mL CuCl_2_·2H_2_O alcohol solution was added. Under the condition of 500 rpm/min, 5 mL of a co-solution containing 0.5% (weight/volume, *w*/*v*) Pluronic F-127 and 10 mM Tris buffer was rapidly added to obtain Rk3/Met-Cu(II) infinite coordination polymers (Rk3/Met-Cu(II) ICPs). Then, 1.5 mg of polydopamine (PDA) was introduced and the mixture was reacted for 24 h in a rotary mixer to ensure sufficient encapsulation. Afterward, the product was lyophilized to obtain PDA@Rk3/Met-Cu(II) nanoparticles (PDA@Rk3/Met-Cu(II) NPs).

### 4.4. Characterization of PDA@Rk3/Met-Cu(II) NPs

Transmission electron microscopy (TEM, Carl Zeiss, Oberkochen, Germany), Zetasizer (Zetasizer Nano, Malvern, Milton Keynes, UK), dynamic light scattering (DLS, Malvern), X-ray photoelectron spectroscopy (XPS, Thermo Scientific Nexsa, Waltham, MA, USA), and Fourier-transform infrared (FT-IR, Nicolet Instrument Corporation, Madison, WI, USA) spectroscopy were employed to characterize the PDA@Rk3/Met-Cu(II) NPs. High-performance liquid chromatography (HPLC, Shimadzu LC-20A, Kyoto, Japan) was utilized to determine the drug release rate under different pH values.

### 4.5. Preparation of Rk3/Met URS MN

Tip formulation: PVA and PVP were mixed at a 5:2 *w*/*w* ratio in deionized water to achieve a combined concentration of 30% *w*/*v*. Subsequently, 2% *w/v* PDA@Rk3/Met-Cu(II) NPs were incorporated into the solution via sonication-assisted loading, followed by magnetic stirring for 30 min to ensure uniform dispersion. Hydrogel backing synthesis: CF-1552-ADH: 3% *w/v* CF-1552 and 0.7% *w/v* ADH were crosslinked using EDC/HOBt (0.15% each) at room temperature (pH 5.5, 24 h), followed by dialysis for 5 days and subsequent lyophilization. Oxidized pullulan (OP): 1% *w/v* pullulan was treated with 5.4 mM NaIO_4_ (2.5 h dark), quenched with ethylene glycol. The solution was then dialyzed for 3 days and lyophilized. CF-1552-ADH and OP were mixed in proportion to obtain the CAOP hydrogel, which served as the MN backing material.

In a typical experiment, the Rk3/Met URS MNs were prepared in two steps using a layer-by-layer curing method. The tip material was centrifugally filled into the upper layer of the needle tips and dried overnight at 37 °C. Then, the backing material was poured. Finally, a complete MN patch was obtained by drying overnight at 37 °C and carefully demolding.

### 4.6. Characterization of Rk3/Met URS MN

Scanning electron microscopy (SEM, Carl Zeiss, Oberkochen, Germany) and Fourier-transform infrared spectroscopy (FT-IR, Nicolet Instrument Corporation, USA) were utilized to characterize the Rk3/Met URS MNs. A MN was placed on the horizontal device of a universal testing machine with the tip facing upward and the force-sensing element was slowly pressed onto the MN to measure the force that a single needle of the MN could withstand. The MN was inserted into the skin and then retrieved at different time points. The remaining height of the MN was measured and SEM was employed to characterize it for the evaluation of its dissolution rate.

### 4.7. Characterization of CAOP Hydrogel Performance

Hydration retention capacity: Hydrogel samples (10 mm diameter × 2 mm thickness) were maintained under ambient conditions (25 °C, 40% RH). Gravimetric analysis was performed daily using an analytical balance (Mettler Toledo ME204E, ±0.1 mg) to determine mass loss. The water loss rate was calculated using the following formula.

(1) $$Weight\;loss\;rate\left(\%\right)=\left(W_{0}-W_{t}\right)/W_{0}\times 100\%$$

where *W_t_* is the weight at time *t* and *W*_0_ is the initial weight of the sample.

The adhesiveness of the CAOP hydrogel was verified via lap shear experiments. A universal testing machine was employed to evaluate the tensile properties of the CAOP hydrogel. Antioxidant capacity assessment: For 1,1-diphenyl-2-picryl-hydrazyl radical (DPPH) radical scavenging, 50 mg hydrogel samples were incubated with 200 µL 0.1 mM DPPH (Sigma-Aldrich, D9132) in ethanol (37 °C, 1 h). Absorbance was measured using a UV–Vis spectrophotometer (Shimadzu UV-2600i). Scavenging activity (%) was calculated at 517 nm. For hydroxyl radical (·OH) scavenging, samples (25 mg) were immersed in Fenton reagent (1 mL containing 2 mM FeSO_4_ + 2 mM H_2_O_2_). After 30 min incubation (37 °C), 100 μL supernatant was reacted with 10 mM TMB (in DMSO) for chromogenic development (650 nm calculate scavenging rate). For hydrogen peroxide (H_2_O_2_) scavenging, 200 mg hydrogel samples were exposed to 0.8 mM H_2_O_2_ (1 mL) for 1.5 h (37 °C). Residual H_2_O_2_ was quantified via the iodide oxidation method, where 150 μL of the reaction mixture was mixed with 150 µL of 1 M KI and the mixture was incubated for 5 min (350 nm calculate scavenging rate). All antioxidant assays included triplicate measurements with appropriate controls (blank: hydrogel + solvent; control: radical solution without hydrogel). The scavenging rate was calculated using the following formula.

(2) $$Scavenging\;rate\left(\%\right)=\left[1-\left(A_{\mathrm{sample}}-A_{\mathrm{blank}}\right)/\left(A_{\mathrm{control}}-A_{\mathrm{blank}}\right)\right]\times 100\%$$

### 4.8. Biocompatibility of MNs

To comprehensively evaluate the blood compatibility of the MNs, an in vitro hemolysis experiment was meticulously carried out. Cytotoxicity was assessed via MTT assay (HUVECs, 72 h exposure) and AO/EB dual staining. The detailed experiment parts are described in the Supplementary Materials.

### 4.9. In Vitro Scratch Assay and Tube Formation Experiments

Human Umbilical Vein Endothelial Cells (HUVECs) were seeded at 1 × 10⁵ cells/well in 6-well plates pre-marked with grid lines on the underside. Cells were maintained in medium containing 10 ng/mL CXCL4 and 10 ng/mL TGF-β under standard culture conditions (37 °C, 5% CO_2_) for 24 h. Monolayers were mechanically disrupted using a 200 µL sterile pipette tip along predefined grid lines. The medium was replaced with fresh serum-free medium and different drug extracts. The groups were divided into the control group, the free Rk3 group, the free Met group, the free Rk3 and Met combination group, and the Rk3/Met URS MN Tip group (with the same amount of Rk3 and Met). The cells were placed in a 37 °C, 5% CO_2_ incubator for 24 h and photographed.

For the tube formation experiments, 30 µL Corning^®^ Matrigel^®^ Growth Factor Reduced (GFR) Basement Membrane Matrix (356230) was polymerized in 24-well plates (37 °C, 30 min). HUVECs (8 × 10^4^ cells/well) were suspended in conditioned medium (10 ng/mL CXCL4 + TGF-β) and added to matrix-coated wells. Experimental groups paralleled scratch assay conditions. Cultures were maintained for 24 h (37 °C, 5% CO_2_). The grouping was the same as that in the cells scratch assay. The cells were imaged using a microscope.

### 4.10. The Anti-Inflammatory and Anti-Fibrotic Effects of Rk3/Met URS MN Tip

Human skin fibroblasts (HSFs) were stimulated with 10 ng/mL CXCL4 and 10 ng/mL TGF-β. The cells were seeded into culture plates and allowed to adhere to the walls. Corresponding drugs were added according to the groups, which were divided into the control group, the free Rk3 group, the free Met group, the free Rk3 and Met combination group, and the Rk3/Met URS MN Tip group (with the same amount of Rk3 and Met). The cells were further cultured for 24 h. Subsequently, the cell-culture supernatants were collected and the levels of IL-6, TNF-α, and Col I were detected using enzyme-linked immunosorbent assay (ELISA) kits.

### 4.11. In Vivo Toxicity of MNs

Major organs (heart, liver, spleen, lung, and kidney) from mice were hematoxylin-eosin staining (H&E staining) following a 10-day MN application.

### 4.12. Efficacy Validation in SSc Mouse Models

Forty female BALB/c mice were randomly allocated into four groups. In the first three groups, a daily injection of 0.1 mL of 0.8 mg/mL bleomycin phosphate-buffered saline (Xiaoyou Biotechnology, Hangzhou, China) was administered into the dorsal region of the mice for four consecutive weeks to induce SSc mouse models. The fourth group was injected with PBS on a daily basis. Subsequently, on the 29th day, three mice were randomly selected from each group for euthanasia to observe the modeling process. The four groups were designated as follows: the normal group consisting of unmodeled mice, the control group containing modeled mice treated with PBS, the free Rk3/Met MN group composed of modeled mice treated with free Rk3 and Met MN, and the Rk3/Met URS MN group of modeled mice treated with Rk3/Met URS MN. The MNs were inserted into the dorsal skin of the mice, and 200 µL of water was sprayed onto the MN backing to restore their hydrogel properties. The MNs were then fixed for a 10-day treatment. All experimental protocols were approved by the Animal Ethics Committee of Northwest University (Protocol Number: NWU-AWC-20240722M, approval date: 11 July 2024).

### 4.13. Histopathological Analysis

Paraffin-embedded sections (4 µm) were stained with H&E and Masson’s trichrome. Immunofluorescence staining was performed for IL-6, TNF-α, Col I, ACA, α-SMA, CD31, TGF-β, and CXCL4.

### 4.14. In Vivo RNA Sequencing and Analysis

On the 10th day, RNA sequencing (RNA-seq) and analysis were performed on the dorsal skin of three mice from the model group and three mice from the Rk3/Met URS MN group.

### 4.15. Statistical Analysis

Data are presented as mean ± standard deviation (SD) in all tests (*n* ≥ 3). Statistical differences between data of various groups were analyzed by one-way ANOVA with Tukey’s post hoc test (GraphPad Prism, version 5.0). Significance thresholds: * *p* < 0.05, ** *p* < 0.01, *** *p* < 0.001, **** *p* < 0.0001.

## Data Availability

The original contributions presented in this study are included in the article/Supplementary Materials. Further inquiries can be directed to the corresponding author.

## Supplementary Materials

The following supporting information can be downloaded at: https://www.mdpi.com/article/10.3390/gels11060384/s1, Figure S1: (a) Particle size statistics of Rk3/Met-Cu(II) ICPs. (b) Particle size statistics of PDA@Rk3/Met-Cu(II) NPs; Figure S2: (a) FT-IR spectrum of CF-1552-ADH. (b) FT-IR spectrum of OP; Figure S3: (a) Statistical results of pore size of hydrogels; Figure S4: (a) Preparation of Rk3/Met URS MN by two-step casting method; Figure S5: (a) Hemolysis rates of different components in Rk3/Met URS MN. (b) Cell viability of HUVECs. (c) AO/EB staining of HUVECs treated with extracts of MNs of different components; Figure S6: (a) Results of total mesh area in each group in the tube formation experiment. (b)Results of total lenght in each group in the tube formation experiment; Figure S7: (a) Fold change to control in the tube formation experiment; Figure S8: (a) H&E staining and Masson staining results. (b) Mice boby weight during modeling process; Figure S9: (a) H&E stained organs revealed no significant signs of toxicity; Figure S10: (a) Recovery of hair growth on the back of mice.
