# Supplementary material for: A Self-Adhesive Ginsenoside Rk3/Metformin-Loaded Hydrogel Microneedle for Management of Systemic Sclerosis
Source: Gels. 2025 May 23;11(6):384. doi: 10.3390/gels11060384 (PMC12192530; doi:10.3390/gels11060384)
Supplement: Supplementary file 1 [file gels-11-00384-s001.zip › gels-3630904-supplementary.pdf]

Supporting information

A self-adhesive ginsenoside Rk3/metformin-loaded hydrogel microneedle for systemic sclerosis: precise transdermal delivery, ultra-long release and dual-target regulation of CXCL4/TGF-β

Yuanyuan Wang[a,b], Caiyun Zhong[a,b], Kexin Wang[a,b], Shihong Shen[,a,b], Daidi Fan[*,a,b]*

a. Engineering Research Center of Western Resource Innovation Medicine Green Manufacturing, Ministry of Education, School of Chemical Engineering, Northwest University, Xi'an, 710127, China

b. Biotech. & Biomed. Research Institute, Northwest University, Xi'an, 710127, China

E-mail: shenshihong@nwu.edu.cn

E-mail: fandaidi@nwu.edu.cn

Tel: 086-029-88305118; Fax: 086-029-88322585

ORCID ID

Shihong Shen ID (0000-0003-1448-9823)

Daidi Fan ID (0000-0001-9798-1674)

Keywords: systemic sclerosis, hydrogel microneedle, dual-target regulation, ultra-long release

S1. Details of Experiment Methods

S1.1 Biocompatibility of MNs

To comprehensively evaluate the blood compatibility of the MNs, an in vitro hemolysis experiment was meticulously carried out. Normal saline was selected as the negative control group, while deionized water served as the positive control group. Subsequently, the absorbance of each sample was precisely measured at a wavelength of 570 nm using a spectrophotometer. The hemolysis rate was calculated by applying the formula.

$$Hemolysis\ rate(\%) = [(A_s - A_n/A_P - A_n]) \times 100\% \quad \text{(S1)}$$

where A_s represents the absorbance value of the sample under test, A_p denotes the absorbance of the positive control, and A_n stands for the absorbance of the negative control. This calculation enables an accurate quantification of the hemolytic potential of the MN.

To further explore the cytotoxicity of the Rk3/Met URS MN, the 3-(4,5-dimethylthiazo1-2-yl)-2,5 -diphenyltetrazolium bromide (MTT) assay was employed. In this assay, HUVECs were seeded in a 96-well plates and incubated with different concentrations of the Rk3/Met URS MN extract for 24 h. Then, MTT solution was added, and after a further incubation, the formazan crystals formed were solubilized, and the absorbance was measured to assess cell viability.

$$\text{Cell viability } (\%) = \left[\left(OD_{sample} - OD_{blank}/OD_{control} - OD_{blank}\right]\right) \times 100\% \quad \text{(S2)}$$

Additionally, acridine orange (AO)/ethidium bromide (EB) double-staining was performed. Cells were treated with the Rk3/Met URS MN extract and then stained

with AO and EB. The stained cells were observed under a fluorescence microscope, allowing for the discrimination between live and dead cells based on the distinct fluorescence patterns. This combination of methods provides a comprehensive evaluation of the cytotoxicity of the Rk3/Met URS MN towards cells.

S2. Supporting Results

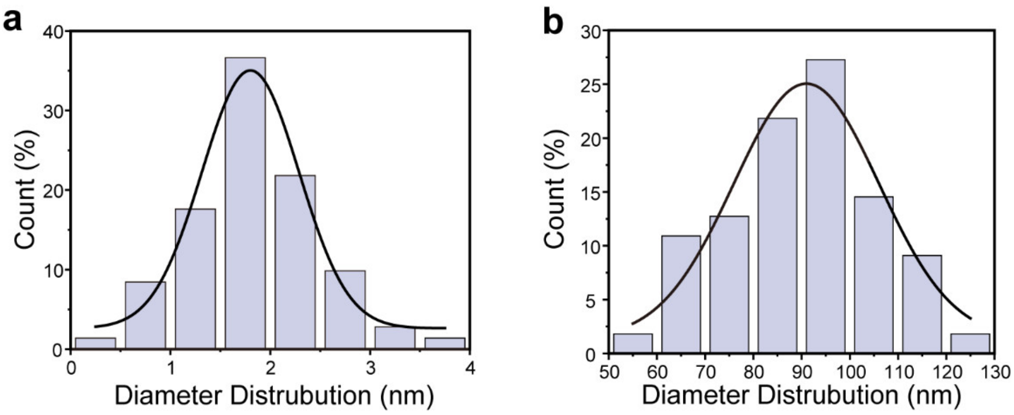

Figure S1. (a) Particle size statistics of Rk3/Met-Cu(II) ICPs. (b) Particle size statistics of PDA@Rk3/Met-Cu(II) NPs.

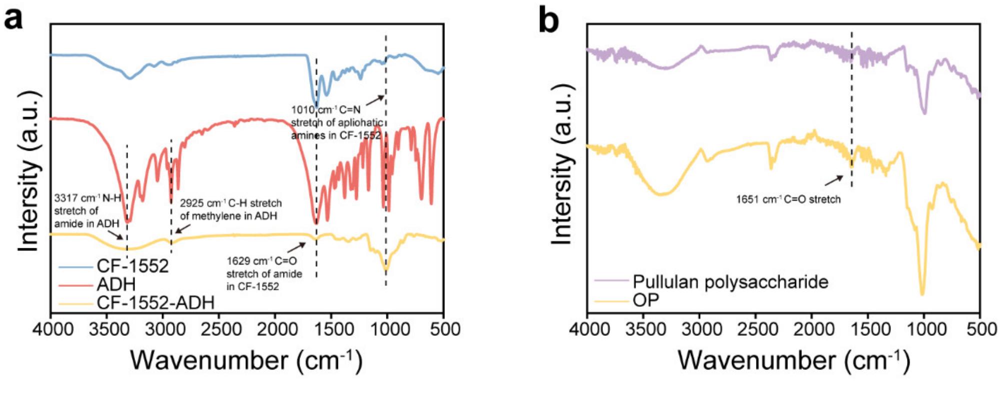

Figure S2. (a) FT-IR spectrum of CF-1552-ADH. (b) FT-IR spectrum of OP.

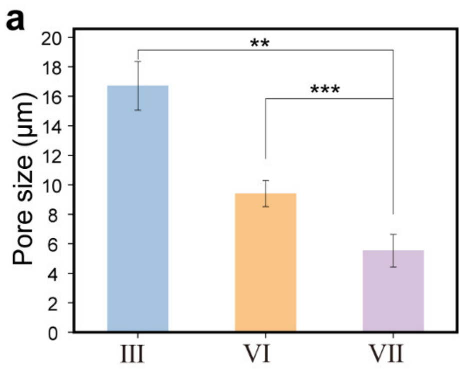

Figure S3. (a) Statistical results of pore size of hydrogels.

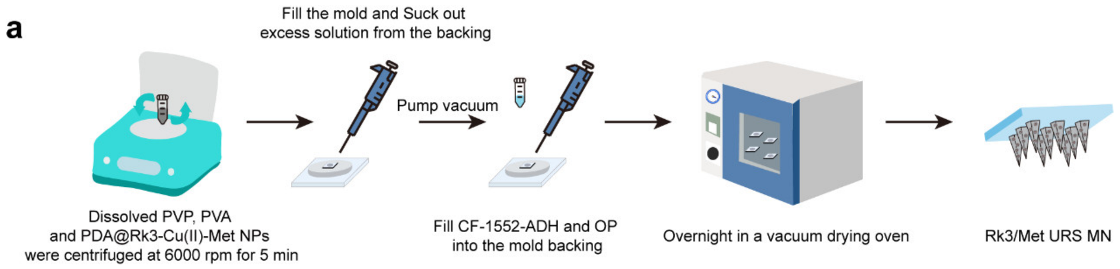

Figure S4. (a) Preparation of Rk3/Met URS MN by two-step casting method.

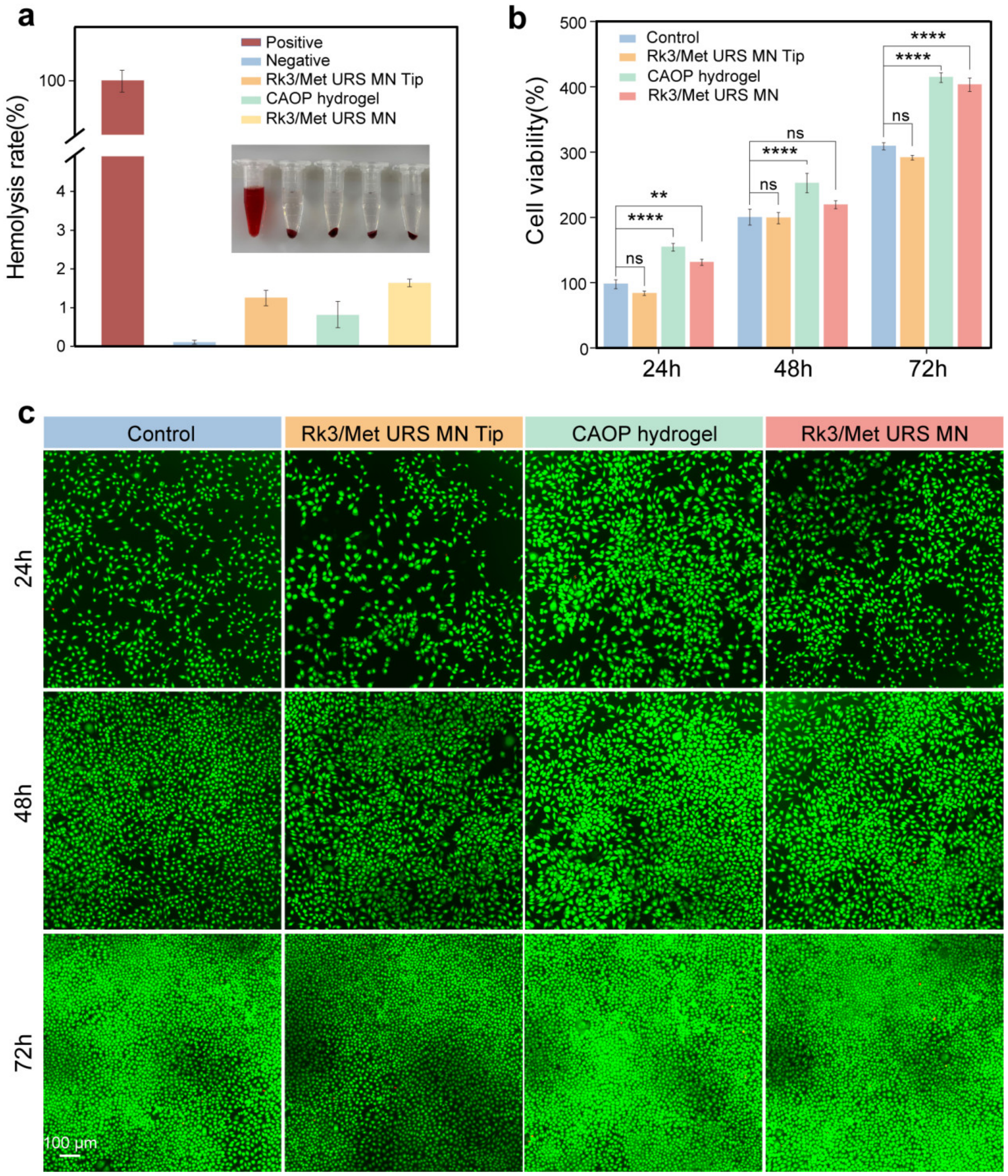

Figure S5. (a) Hemolysis rates of different components in Rk3/Met URS MN. (b) Cell viability of HUVECs. (c) AO/EB staining of HUVECs treated with extracts of MNs of different components.

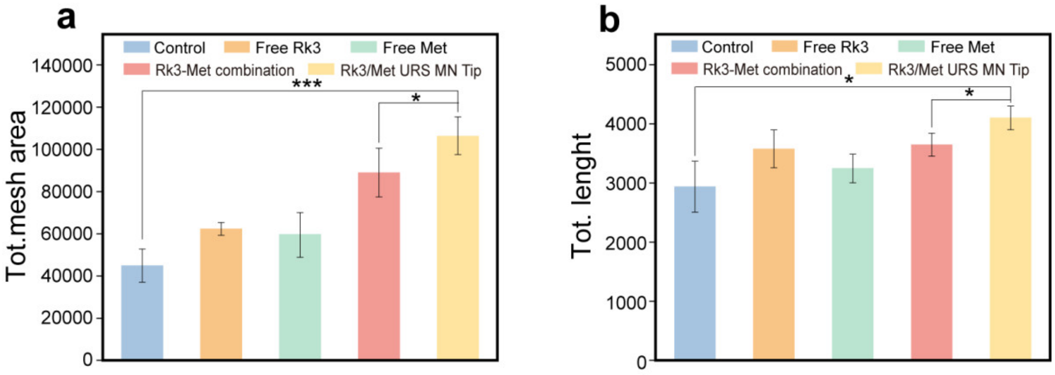

Figure S6. (a) Results of total mesh area in each group in the tube formation experiment. (b)Results of total lenght in each group in the tube formation experiment.

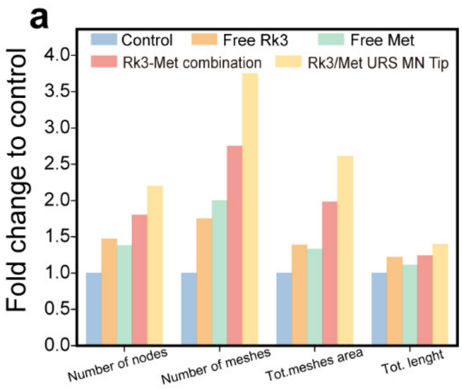

Figure S7. (a) Fold change to control in the tube formation experiment.

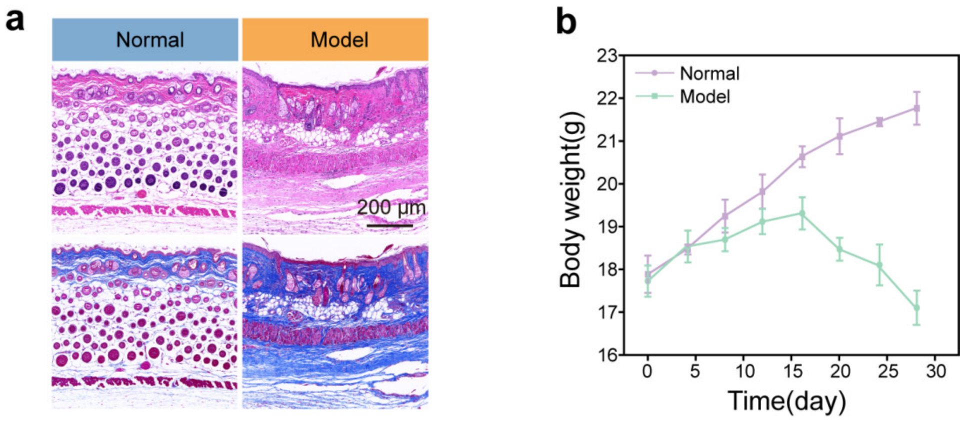

Figure S8. (a) H&E staining and Masson staining results. (b) Mice boby weight during modeling process.

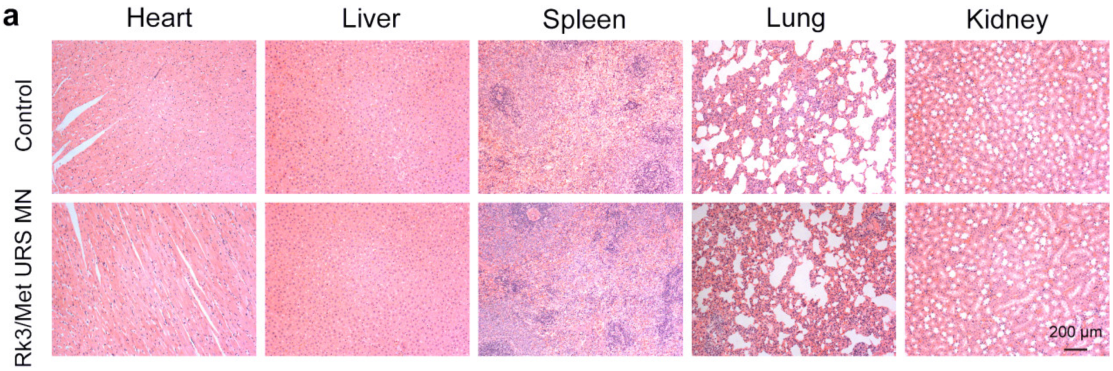

Figure S9. (a) H&E stained organs revealed no significant signs of toxicity.

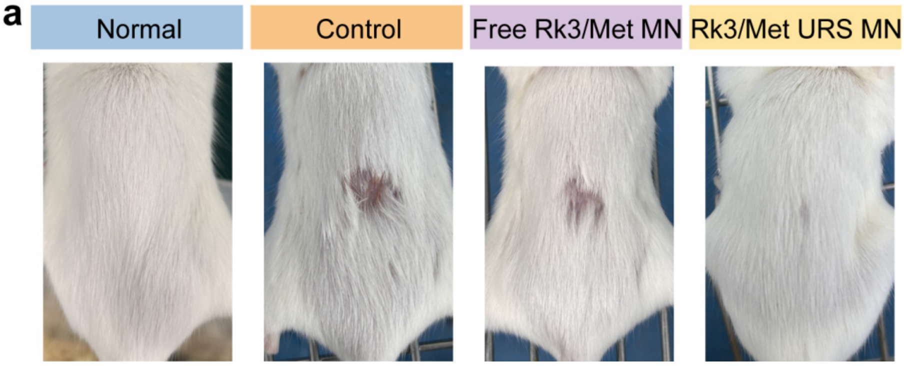

Figure S10. (a) Recovery of hair growth on the back of mice.
